# Artificial intelligence in the psychologist’s toolkit: Psypilot as a case study

**DOI:** 10.3389/fpsyg.2026.1775464

**Published:** 2026-02-27

**Authors:** Pablo Roca, Rosaria Maria Zangri, Guillermo Rodriguez-Fernandez, Martin Sanchez-Pedreño, Eduardo P. García del Valle

**Affiliations:** 1 Precision Mind Lab (Premind), Faculty of Health Sciences, Universidad Villanueva, Madrid, Spain; 2 Medea Lab, Madrid, Spain; 3 Facultad de Salud, UNIE Universidad, Madrid, Spain; 4 School of Science and Technology, IE University, Madrid, Spain

**Keywords:** AI, artificial intelligence, clinical decision support, large language models, measurement-based care, precision mental health

## Abstract

Artificial intelligence (AI) is rapidly reshaping how psychology is practiced, from assessment and case formulation to intervention planning, monitoring, and documentation. Yet the field faces a strategic choice: deploy AI as a substitutive “automated therapist,” or develop AI copilots that augment psychologists’ judgment while preserving the relational and ethical core of professional work. In this article, we synthesize how contemporary AI-especially Machine Learning and Large Language Models- maps onto psychologists’ core tasks and discuss the implications for clinical quality, scalability, and innovation in real-world settings. We then present Psypilot as a case study of the copilot paradigm: an AI-powered clinical assistance platform designed to support Precision Mental Health. We critically examine key risks and governance challenges such as automation bias, data representativeness and fairness, privacy and secondary use, transparency, and accountability under emerging regulatory frameworks, and translate them into practical design and training recommendations. By framing AI as workflow-embedded decision support rather than autonomous care, this contribution advances responsible innovation and clarifies the competencies psychologists need to thrive in an AI-driven professional landscape.

## Introduction

Over the past decade, mental health has moved from being a “silent pandemic” to a clearly quantified global crisis. The latest World Health Organization mental health report (World Health Organization, 2025) estimates that more than one billion people—14% of the global population and over one in eight individuals worldwide- are currently living with a mental health condition. Mental disorders account for around one in 6 years lived with disability (YLDs) globally, placing them among the leading causes of disability across the life course. The economic impact is equally striking: depression and anxiety alone cost the global economy an estimated US$ 1 trillion per year in lost productivity, and broader estimates of the costs of poor mental health point to multi-trillion-dollar losses annually.

Despite this burden, responses remain markedly insufficient, with persistent gaps in funding, specialist workforce and quality of care. Governments still allocate around 2% of health budgets to mental health, and median mental-health workforce levels remain extremely low in many countries (World Health Organization, 2025). A 2025 systematic review of service coverage in Europe found that large proportions of adults with major depression, bipolar disorder or psychotic disorders are not receiving minimally adequate care, with average treatment gaps of around 45% (Barbui et al., 2025). These figures are not just a problem of low- and middle-income countries, and they reflect an inability of current mental health service models to scale to real demand.

For psychologists working in real-world settings (health, organizational, educational and others), these figures translate into very concrete pressures: heavy caseloads, long waiting lists, expanding documentation and reporting requirements, and growing demands for prevention and well-being initiatives in workplaces and schools (OECD, 2022). In this context of structural constraints (time, workforce, budgets), doing more of the same is unlikely to be sufficient. It is therefore necessary to develop innovative solutions and explore technological tools that can help identify needs earlier and more efficiently, personalize interventions, and reduce low-value administrative tasks so that professional time can be focused on high-value relational and conceptual work. This is the backdrop against which Artificial Intelligence (AI) becomes relevant for psychology: not as a trend, but as a necessary response to the persistent mismatch between mental health needs and available resources (Lee et al., 2021).

## The rise of AI in healthcare

While AI in mental health is still at an early stage of implementation, in the medical field AI systems have already progressed from experimental prototypes to tools that match or surpass specialist performance (Morone et al., 2025). For instance, AI models have achieved dermatologist-level performance in classifying skin cancer and, in some analyses, have outperformed the average board-certified dermatologist in distinguishing malignant from benign lesions (Esteva et al., 2017). Beyond image interpretation, autonomous AI systems for diabetic retinopathy screening in youth increased screening completion and follow-up compared with traditional referral pathways, demonstrating not only diagnostic accuracy but also real-world impact on care processes (Wolf et al., 2024). Taken together, these examples support three points that are highly relevant for psychology: (1) AI can reach—and sometimes exceed—specialist-level performance when tasks are well defined, data are plentiful and outcomes are clearly measurable; (2) The most successful deployments are integrated into clinical workflows as Clinical Decision Support Systems (CDSS), not as stand-alone “black boxes.” Clinicians need to understand what a model does, what input it uses, how reliable it is and how to challenge its outputs, rather than simply accepting algorithmic recommendations; and (3) Regulatory and ethical frameworks are emerging that treat AI not as a replacement for clinicians, but as a new class of medical device whose performance, safety and equity must be empirically evaluated.

In mental health, analogous developments are now underway. AI is promoting a paradigm shift, helping us implement Precision Mental Health (Bickman, 2020). Applying the same principles as Precision Medicine (Sahu et al., 2022), precision mental health aims to deliver the right intervention, at the right intensity, at the right time, by the right clinician, for a given individual, using data to inform these choices in a systematic way, rather than relying solely on averages or intuition. Reviews in clinical psychology and psychiatry show a rapidly growing literature on AI models that support diagnosis, prognosis and treatment selection (Dwyer et al., 2018). Applications span risk prediction, symptoms trajectory modeling, relapse detection and digital phenotyping, as well as the use of NLP on clinical notes, therapy transcripts and social media data (Shatte et al., 2019).

## AI across the psychologist’s core tasks

**Figure 1:**
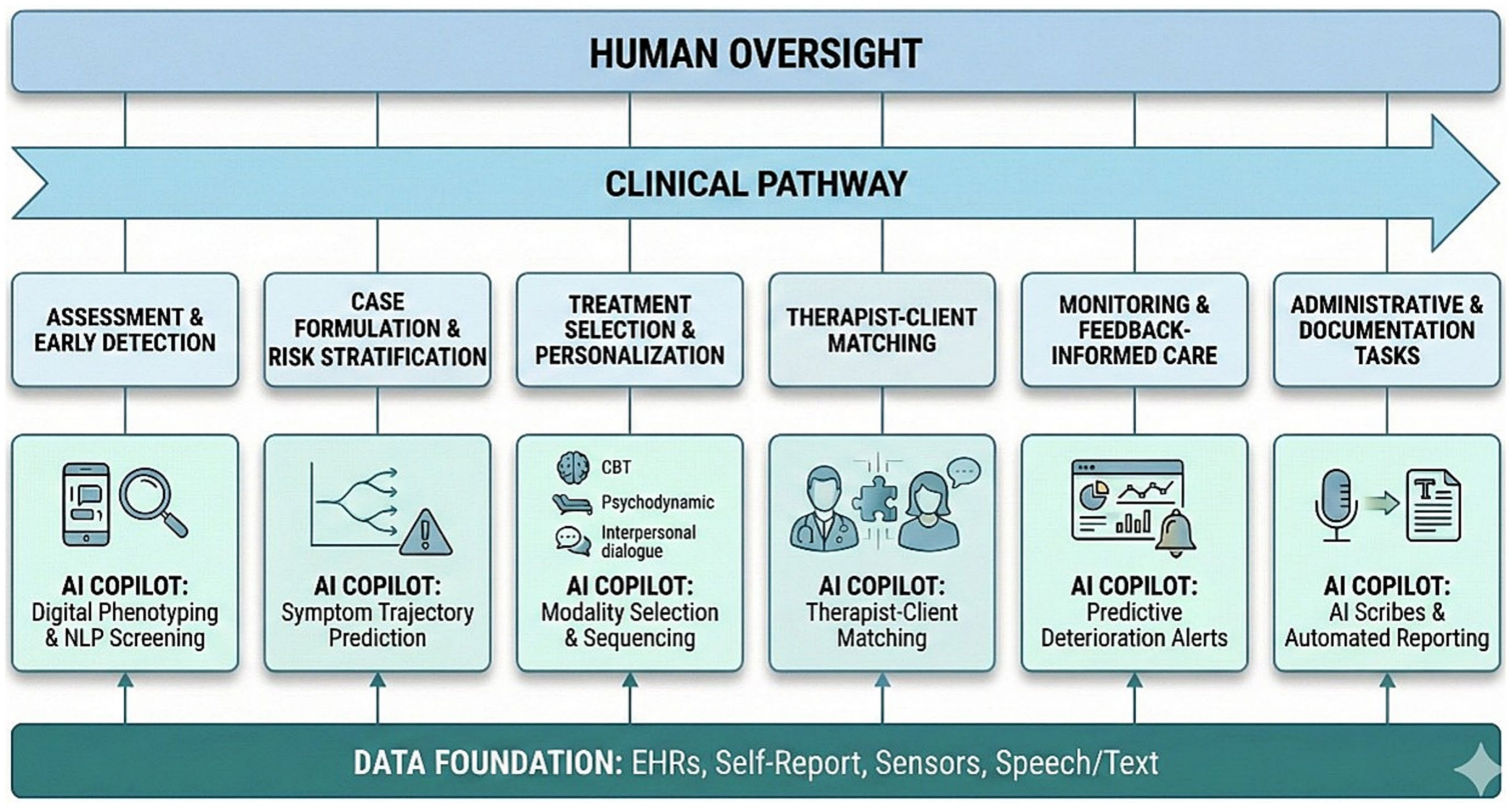
AI Across the psychologist’s core tasks.

A useful way to think about AI in psychology is as a set of tools that map onto core professional tasks. AI technologies are beginning to touch every major task in the psychologist’s workflow, from how we assess, to how we decide on and deliver interventions, and how we monitor and document care (see Figure 1):

- *Assessment and early detection*: most ML studies have focused on detecting or classifying mental health conditions (especially depression, anxiety, psychosis and suicidality) using clinical, self-report and digital data, showing that AI tools are generally accurate in detecting, classifying and predicting the risk of mental health conditions (Leaning et al., 2025; Spittal et al., 2025). Three assessment trends are particularly relevant for psychologists: (1) Enhanced screening from routine data: AI models trained on demographics, questionnaires, EHR, and clinical variables can distinguish individuals with depression or anxiety from healthy controls with reasonable accuracy, sometimes outperforming traditional scoring rules; (2) Digital phenotyping and passive monitoring: Studies using smartphone and wearable data (e.g., mobility patterns, sleep regularity, communication frequency) show that ML can detect changes associated with mood episodes, relapse risk or deterioration, sometimes days before traditional assessments would detect them; and (3) Language and voice as assessment signals: linguistic markers in clinical notes, therapy transcripts and social media can be used to classify depression, PTSD, psychosis and suicidal ideation, and to track symptom change over time.
- *Case formulation and risk stratification*: AI is increasingly used to help answer “what is likely to happen if we do nothing, or if we intervene?”. Several included studies used AI to predict future symptom trajectories, relapses, hospitalization or treatment response, rather than just baseline diagnosis (Kusuma et al., 2024). For psychologists, these tools could act as structured extensions of case formulation: instead of solely relying on qualitative judgment to estimate risk, we can consult models trained on thousands of similar cases and integrate their output (with appropriate caution) into our understanding.
- *Treatment selection and personalization*: perhaps the most attractive and challenging promise of AI in psychology is that it might help answer the question: “Which intervention is most likely to benefit this particular client?”. ML models can predict response vs. non-response to antidepressants or psychotherapy with moderate accuracy, especially when they incorporate multimodal data (Lutz et al., 2025). For psychological treatments specifically, several lines of work are relevant: (1) Predicting who will benefit from evidence-based therapies; (2) Modality selection (e.g., CBT vs. psychodynamic therapy); (3) Adaptive sequencing and stepped care (e.g., stepping up intensity when early response is poor, switching modality when risk of non-response is high, or extending treatment when relapse risk is elevated).
- *Patient-therapist matching*: The first critical decision that patients and clinicians face occurs even before treatment begins: “Who is the most suitable therapist for this patient?”. Traditionally, patients are matched with therapists based on administrative criteria, scheduling availability or geographic proximity. However, a growing body of research highlights that therapists achieve varying average outcomes with their patients, a phenomenon known as the Therapist Effect (Owen et al., 2015). The Therapist Effect can be categorized into two components: (1) Between-therapist effects, which refer to differences in average treatment outcomes across therapists (Johns et al., 2019); and (2) Within-therapist effects, where individual therapists demonstrate greater effectiveness with certain types of patients or disorders and less effectiveness with others. Empirical studies suggest substantial differences in patient improvement rates and adherence when using empirical matching strategies compared to standard case assignment procedures (Constantino et al., 2020). AI models are being developed to predict the potential fit between a specific patient and therapist, analyzing structured data (e.g., client symptoms, demographic information for both patients and therapists) alongside empirically derived data on therapist performance strengths.
- *Monitoring and feedback-informed care*: Monitoring symptoms, functioning, alliance and other process variables over time is central to good psychological practice, but difficult to maintain consistently in busy services. Measurement-based care (MBC) and feedback-informed treatment aim to address this by integrating brief, repeated measures into routine care and feeding that information back to clinicians and clients (Lutz et al., 2022). AI contributes here in at least two ways: (1) Predictive monitoring: AI models could provide an early signal of who is at risk of deteriorating or not improving, or to predict dropout, non-response or relapse based on early in-treatment change patterns, allowing clinicians to intervene sooner; and (2) Smart feedback and visualization: data-informed systems can automatically generate dashboards that show individual trajectories against expected benchmarks or visualize changes in process variables (e.g., alliance, adherence, motivation). From a psychologist’s viewpoint, this moves us from retrospective-impressionistic monitoring (“I think things are improving”) to prospective data-informed monitoring, where both therapist and client can see patterns and adjust collaboratively.
- *Administrative and documentation tasks*: AI may have pragmatic value for psychologists by reducing administrative and documentation burden. Preliminary industry reports and early academic studies have suggested that, in certain contexts, AI scribes can reduce time spent on notes by 40–60%, and may improve the completeness and structure of documentation, potentially freeing clinicians to focus more on direct patient interaction and self-care (Olson et al., 2025).

## Psychology at a crossroads

The question for psychology is not whether AI can achieve clinically relevant performance -it clearly can- but how we want these systems to be positioned in relation to professional practice (Roca, 2025). Psychology faces a strategic and ethical choice between two broad approaches for how AI is implemented in practice: (1) Substitutive AI (i.e., AI-Based Therapy), which involves the use of AI to fully automate the therapeutic process, replacing the professional with therapeutic chatbots that interact with the patient by simulating human behavior; and (2) AI copilots (i.e., AI-Assisted Therapy), which involves the use of AI to complement and enhance the work of the professional, rather than replace them.

In the AI substitutive model, the user interacts directly with AI systems -typically chatbots or self-guided apps- without active involvement of a human psychologist. The logic is compelling from a public-health and business standpoint: massive scalability (24/7 access, virtually unlimited “slots”), very low marginal cost per user, and potential to reach populations who would never attend traditional services. AI-based chatbots are already being used for screening, self-management of symptoms and behavioral change, often with promising but still preliminary evidence on engagement and outcomes (Boucher et al., 2022). However, this substitutive approach raises significant concerns: psychologists may be bypassed in favor of direct-to-consumer AI platforms, when something goes wrong it is unclear who is responsible (developers, providers, users), data privacy, algorithmic bias against minoritized groups, and the risk of users developing maladaptive attachments or over-reliance on AI agents (Le Glaz et al., 2021). Therefore, although substitutive AI may help close some access gaps, it also risks creating a parallel mental health ecosystem in which psychological expertise is embedded in code and business models rather than in accountable professionals.

An alternative is to conceive AI as a copilot for psychologists: a set of tools that augment professional judgment and therapeutic relationship rather than replace it. From this perspective, AI systems are embedded within the psychologist’s workflow to synthesize information from Electronic Health Records (EHRs), questionnaires, interviews, or digital traces into decision-support outputs (e.g., triage, treatment selection, prognosis, monitoring). Copilots also reduce administrative burden by assisting with notetaking, report drafting, and documentation, as well as providing ongoing monitoring and feedback that can be integrated into supervision, case formulation, and shared decision-making with clients (Dwyer et al., 2018). The psychologist remains primarily responsible, interpreting AI-copilot outputs considering contextual knowledge, ethics, and the client’s narrative. Governance, training, and evaluation focus on how the AI copilot changes the quality and equity of human-delivered care. Copilots are treated as a fallible but useful source of structured information—analogous to a GPS in driving—rather than as an autonomous therapist. The distinction between prediction and decision is exactly where the idea of AI as a “copilot” for psychologists becomes concrete: AI is not deciding what to do with a client; it is helping us see more clearly what might happen under different options, so that our decisions can be more transparent, data-informed and individualized.

## Case example: Psypilot as a AI copilot for psychologists

Building on this copilot perspective, Psypilot provides a case example of how a copilot approach may be operationalized within routine psychological practice. Psypilot was explicitly designed as an AI for psychologists rather than instead of psychologists: a professional-grade tool that aims to make assessment, decision-making, treatment planning and monitoring more precise and efficient, while leaving the relational, ethical and interpretive core of psychological work where it belongs: with the human professional.

Psypilot is an AI-powered clinical assistance platform designed specifically for psychologists and mental health services to implement Precision Mental Health, promoting measurement-based care and data-driven decision-making. Psypilot facilitates the use of established psychological evaluation tools with robust psychometric support, e.g., brief symptom measures such as the Patient Health Questionnaire-9 (PHQ-9) (Kroenke et al., 2001) for depressive symptoms and the Generalized Anxiety Disorder-7 (GAD-7) (Spitzer et al., 2006). In addition, it incorporates a novel multifactorial screening instrument designed to capture a broader biopsychosocial profile and support precision-oriented care. This instrument has undergone internal validation and is currently under peer review for publication. The multifactorial screening covers, in addition to core symptom domains, key areas related to well-being, lifestyle, and contextual/environmental factors (e.g., functioning and quality-of-life indicators, sleep and health behaviors, and relevant psychosocial factors). Together, these inputs are used to structure the initial clinical picture and to support downstream workflow functions such as personalization, monitoring and progress feedback. However, although Psypilot was developed through scientific translation and its components are grounded in literature, the integration of these elements into a single end-to-end platform and the associated personalization logic must be evaluated independently in routine psychological practice settings.

**Figure 2:**
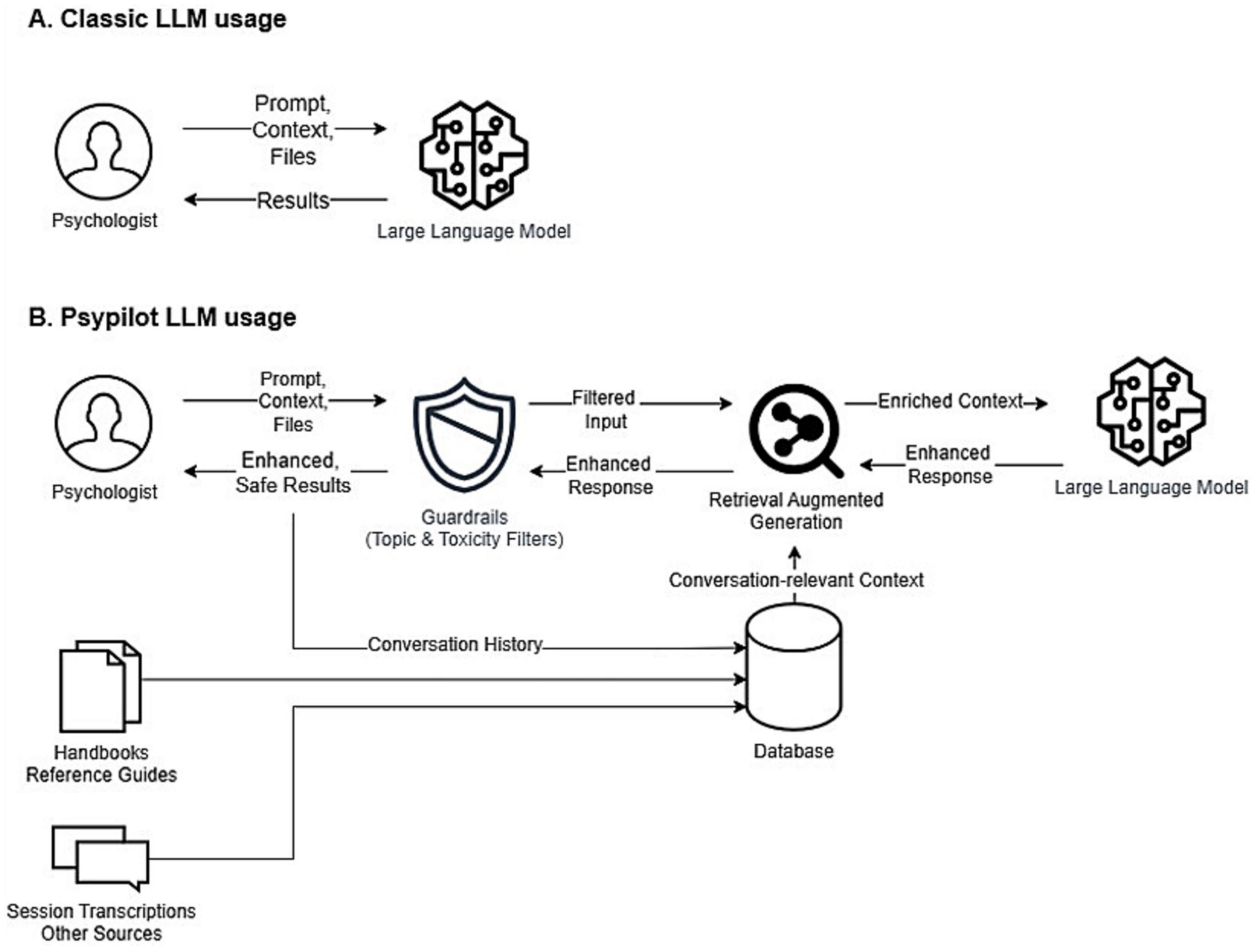
Psypilot as an example case study.

In contrast to general-purpose conversational assistants, which are typically not designed or governed as clinical systems, Psypilot is designed to address common safety and context limitations when large language models are used in mental health workflows (Lawrence et al., 2024). First, it incorporates guardrails intended to constrain interactions to practice-relevant content and to reduce unsafe or toxic outputs (e.g., abusive, discriminatory, or self-harm–promoting content). Second, Psypilot augments model context using Retrieval-Augmented Generation (RAG): relevant elements of the ongoing case context are used to compute embeddings (vector representations) that retrieve semantically related information from a vector database. In the current implementation, this database is built from expert-curated materials (e.g., clinical guidelines) and may also include practice-generated information such as de-identified case history and clinician-provided session notes or transcripts (see Figure 2). This approach is intended to reduce the likelihood of unsupported outputs by grounding responses in retrieved sources, consistent with emerging evidence that retrieval/grounding strategies can mitigate hallucination in health-information chatbots (Nishisako et al., 2025), while also improving transparency by surfacing references that clinicians are encouraged to verify.

Furthermore, Psypilot was designed from the outset to operate within the legal and ethical constraints typically expected in psychological practice, with implementation choices informed by emerging EU governance frameworks for AI-enabled health technologies (Aboy et al., 2024). In practice, this includes data-protection measures such as EU-based cloud regions and EU-hosted model providers, encryption in transit and at rest, and data-minimization principles. In line with the EU AI Act’s emphasis on human oversight for higher-risk applications, Psypilot implements a human-in-the-loop workflow in which outputs are presented as draft decision support subject to clinician review and verification, and relevant system events are logged to support traceability. Crucially, while patient-provided information may be used to enrich case context within the active clinical workflow, it is isolated from model development and is not used to train or improve the underlying models.

## Discussion

The same features that make AI attractive for mental healthcare also create distinctive ethical and regulatory challenges. In a field where the “raw material” of practice is highly personal, sensitive, and often stigmatized experience, these risks are amplified. Consistent with the perspective nature of this article, this section addresses not only opportunities but also limitations, failure modes, and priorities for independent evaluation and future development. Recent reviews converge on several recurring domains of concern (D’Alfonso, 2020; Meadi et al., 2025):

### Risk and safety

Technical risks include model error, instability, adversarial attacks, and loss of control when systems drift over time or are deployed outside their validated context. Clinical risks include misclassification, inappropriate suggestions, or failure to detect acute risk (e.g., suicidality), particularly when AI is used for triage or crisis-related support. A central concern is automation bias: clinicians may over-rely on AI outputs, especially when systems present quantitative confidence scores or are framed as “smart” or “evidence-based” (Shatte et al., 2019). To mitigate these risks, clinical copilots should operationalize human oversight through interface and workflow design—for example, presenting outputs as non-authoritative drafts, requiring active clinician confirmation or editing for clinically consequential content, and embedding clear escalation pathways for high-risk scenarios.

### Regulation and accountability

The regulatory landscape for AI in mental health is rapidly evolving and remains fragmented. In the European Union, the AI Act introduces a risk-based framework that classifies AI systems and imposes stringent requirements on high-risk applications, potentially including clinical decision-support and some patient-facing mental health tools depending on intended use and deployment context (Tavory, 2024). In the UK, a recent parliamentary briefing maps how AI mental health tools fall under a complex mosaic of regulators and standards (e.g., MHRA, NICE, CQC, data protection and equality law), highlighting gaps around responsibility for safety and effectiveness when tools are integrated into routine care (Gardiner and Mutebi, 2025). In the US context, legal scholars debate whether conversational therapy chatbots should be regulated more like licensed telehealth professionals, medical devices, or wellness products, arguing for clearer federal oversight and alignment with existing medical device pathways (Mello and Cohen, 2025). A consistent theme across these analyses is that, even when AI systems play a substantial role in assessment or intervention, professional responsibility for clinical decisions remains with clinicians. Accordingly, AI systems used in psychological practice should be framed as clinician-controlled decision support rather than autonomous practitioners, which foregrounds practical questions about standard of care: What constitutes reasonable reliance on an algorithm? How should clinicians document the role of AI in clinical decision-making?

### Bias and fairness

Systematic reviews of ML in psychiatry and psychotherapy emphasize the limited representativeness of training datasets, which are often small, geographically restricted, and skewed toward White and higher-income populations (Shatte et al., 2019; Aafjes-van Doorn et al., 2021). Models trained on such data risk differential performance across demographic groups, potentially exacerbating existing inequities in diagnosis, treatment allocation, and access to care. Proposed best practices include careful curation of diverse datasets, routine subgroup performance analysis, bias audits, and transparency about known limitations so that clinicians and organizations can interpret outputs appropriately.

### Transparency and explainability

Most high-performing AI systems in mental health (e.g., deep learning and LLMs) are opaque to end-users, raising concerns about explainability and trust. Clinical ethics frameworks traditionally emphasize reason-giving, informed consent, and shared decision-making; yet it is often difficult to provide intelligible explanations for complex model outputs, especially when systems are proprietary (Lee et al., 2021; Mello and Cohen, 2025). Recent reviews recommend a combination of strategies, including using inherently interpretable models for high-stakes decisions when possible, augmenting black-box models with post-hoc explanations (e.g., feature importance, example-based explanations), documenting data sources, and clearly labeling AI-generated content (Putica et al., 2025).

### Governance implications under the EU AI Act

Beyond general ethical principles, the EU AI Act offers a concrete governance template for AI systems used in health-related contexts, particularly when systems may influence decisions affecting health or fundamental rights (Aboy et al., 2024). Complementing this regulatory lens, a recent systematic review of generative AI in mental health synthesized recurrent ethical concerns and proposed an integrative framework (GenAI4MH) structured around four domains: data privacy and security, information integrity and fairness, user safety, and ethical governance and oversight (Wang et al., 2025). Together, these perspectives help translate high-level principles into implementable requirements for clinical copilots in psychological care. Practically, this supports a governance “minimum set” aligned with the AI Act’s emphasis on meaningful human oversight and accountability: (1) explicit intended-use statements and exclusions, including foreseeable misuse scenarios; (2) operationalized human oversight (e.g., outputs framed as drafts, clinician confirmation for consequential content, and clear escalation pathways); (3) traceability through audit logs, model/version tracking, and documentation of retrieved sources where applicable; (4) data governance (minimization, access controls, encryption, and retention limits); (5) pre-deployment evaluation and safety testing (including edge-case/adversarial prompts) with subgroup and language checks to address fairness; and (6) post-deployment monitoring, incident reporting, and iterative improvement processes proportional to risk and context (Wang et al., 2025; Aboy et al., 2024).

Taken together, these domains are not independent challenges but mutually reinforcing: safety failures can be amplified by opacity, bias can be obscured by limited explainability, and fragmented accountability can undermine meaningful clinician oversight. Accordingly, the central implication is practical rather than purely conceptual: risk mitigation requires governance mechanisms that operationalize these concerns into concrete requirements for design, evaluation, documentation, and monitoring. For psychologists, the implication is that adopting AI copilots should be coupled with explicit oversight responsibilities: treating outputs as non-authoritative drafts, verifying sources and clinical reasoning, and ensuring that local governance (privacy, documentation, incident reporting, and escalation procedures) is in place before integrating such tools into routine care.

## Data Availability

The original contributions presented in the study are included in the article/supplementary material, further inquiries can be directed to the corresponding author.

## References

[ref1] Aafjes-van Doorn K. Kamsteeg C. Bate J. Aafjes M. (2021). A scoping review of machine learning in psychotherapy research. Psychother. Res. 31, 92–116. doi: 10.1080/10503307.2020.1808729, 32862761

[ref2] Aboy M. Minssen T. Vayena E. (2024). Navigating the EU AI act: implications for regulated digital medical products. NPJ Digital Med. 7:237. doi: 10.1038/s41746-024-01232-3, 39242831 PMC11379845

[ref3] Barbui C. Alonso J. Chisholm D. Evans-Lacko S. Keynejad R. C. Lazerini M. . (2025). Mind the gap: global and regional unmet need for mental health services. Lancet Reg. Health Eur. 57:101458. doi: 10.1016/j.lanepe.2025.101458 41132773 PMC12541639

[ref4] Bickman L. (2020). Improving mental health services: a 50-year journey from randomized experiments to artificial intelligence. Admin. Pol. Ment. Health 47, 795–843. doi: 10.1007/s10488-020-01065-8, 32715427 PMC7382706

[ref5] Boucher E. M. Harake N. R. Ward H. E. Stoeckl S. E. Vargas J. Minkel J. . (2022). Artificial intelligence in mental health apps: an overview of recent advances. J. Med. Devices 18, 37–49. doi: 10.1080/17434440.2021.2013200 34872429

[ref6] Constantino M. J. Boswell J. F. Coyne A. E. Swales T. P. Kraus D. R. (2020). Therapists as responders: routine outcome monitoring and algorithmic feedback in psychotherapy. Psychother. Res. 30, 801–815. doi: 10.1080/10503307.2019.1614745

[ref7] D’Alfonso S. (2020). AI in mental health. Curr. Opin. Psychol. 36, 112–117. doi: 10.1016/j.copsyc.2020.04.005, 32604065

[ref8] Dwyer D. B. Falkai P. Koutsouleris N. (2018). Machine learning approaches for clinical psychology and psychiatry. Annu. Rev. Clin. Psychol. 14, 91–118. doi: 10.1146/annurev-clinpsy-032816-045037, 29401044

[ref9] Esteva A. Kuprel B. Novoa R. A. Ko J. Swetter S. M. Blau H. M. . (2017). Dermatologist-level classification of skin cancer with deep neural networks. Nature 542, 115–118. doi: 10.1038/nature21056, 28117445 PMC8382232

[ref10] Gardiner H. Mutebi N. (2025). AI and mental healthcare – ethical and regulatory challenges. Lancet Psychiatry 12, 435–437. doi: 10.1016/S2215-0366(25)00058-9

[ref11] Johns R. G. Barkham M. Kellett S. Saxon D. (2019). A systematic review of therapist effects: a critical narrative update and refinement to Bergin and Garfield's review. Clin. Psychol. Rev. 69, 67–82. doi: 10.1016/j.cpr.2018.08.005, 30442478

[ref12] Kroenke K. Spitzer R. L. Williams J. B. W. (2001). The PHQ-9: validity of a brief depression severity measure. J. Gen. Intern. Med. 16, 606–613. doi: 10.1046/j.1525-1497.2001.016009606.x, 11556941 PMC1495268

[ref13] Kusuma K. Larsen M. Quiroz J. C. Gillies M. Burnett A. Qian J. . (2024). Artificial intelligence for personalizing psychological interventions: a scoping review. Nat. Digit. Med. 7:12. doi: 10.1038/s41746-024-00979-4

[ref14] Lawrence H. R. Schneider R. A. Rubin S. B. Matarić M. J. McDuff D. J. Mataric M. J. (2024). A systematic review of conversational agents for mental health: effectiveness, design, and implementation. Nat. Hum. Behav. 8, 221–237. doi: 10.1038/s41562-023-01733-8

[ref15] Le Glaz A. Haralambous Y. Kim-Dufor D.-H. Lenca P. Billot R. Ryan T. C. . (2021). Machine learning and natural language processing in mental health: systematic review. J. Med. Internet Res. 23:e15708. doi: 10.2196/15708, 33944788 PMC8132982

[ref16] Leaning I. E. Ikani N. Savage H. S. Leow A. Beckmann C. Ruhé H. G. . (2025). Generative AI for mental health care: a systematic review and a research agenda. Nat. Ment. Health 3, 218–231. doi: 10.1038/s44220-025-00330-4

[ref17] Lee E. E. Torous J. De Choudhury M. Depp C. A. Graham S. A. Kim H.-C. (2021). Artificial intelligence for mental health: clinical applications, challenges, and directions. Biol. Psychiatry Cogn. Neurosci. Neuroimaging, 6, 856–864. doi: 10.1016/j.bpsc.2021.02.001 33571718 PMC8349367

[ref001] Lutz W. Schwartz B. Delgadillo J. (2022). Measurement-based and data-informed psychological therapy. Annu. Rev. Clin. Psychol. 18, 71–98. doi: 10.1146/annurev-clinpsy-071720-014821, 34910567

[ref002] Lutz W. Schwartz B. Vehlen A. Eberhardt S. T Delgadillo J. (2025). Advances in personalization of psychological interventions. World Psychiatry 24, 343–345. doi: 10.1002/wps.21342, 40948053 PMC12434349

[ref20] Meadi M. R. Sillekens T. Metselaar S. van Balkom A. Bernstein J. Batelaan N. (2025). Large language models in mental health: pitfalls and prospects. Front. Psych. 16:1384015. doi: 10.3389/fpsyt.2025.1384015

[ref21] Mello M. M. Cohen I. G. (2025). Regulation of health and health care artificial intelligence. N. Engl. J. Med. 392, 981–986. doi: 10.1056/NEJMra2408045 40095599

[ref22] Morone G. De Angelis L. Martino Cinnera A. Carbonetti R. Bisirri A. Ciccone M. . (2025). Artificial intelligence in digital mental health: a narrative review. Front. Digit. Health 7:1550731. doi: 10.3389/fdgth.2025.1550731 40110115 PMC11920125

[ref23] Nishisako S. Higashi T. Wakao F. (2025). Reducing hallucinations and trade-offs in responses in generative AI chatbots for cancer information: development and evaluation study. JMIR Cancer 11:e70176. doi: 10.2196/70176, 40934488 PMC12425422

[ref24] OECD (2022). Promoting health and well-being at work: Policy and practices. Paris, France: OECD Publishing.

[ref25] Olson K. D. Meeker D. Troup M. Barker T. D. Nguyen V. H. Manders J. B. . (2025). Generative AI for clinical documentation: a randomized trial. JAMA Netw. Open 8:e2534976. doi: 10.1001/jamanetworkopen.2025.34976 41037268 PMC12492056

[ref26] Owen J. Drinane J. M. Idigo K. C. Valentine J. C. (2015). Psychotherapist effects in meta-analyses: how accurate are treatment effects? Psychotherapy 52, 268–277. doi: 10.1037/pst0000021, 26301423

[ref27] Putica A. Khanna R. Bosl W. Saraf S. Edgcomb J. (2025). Ethical decision-making frameworks for AI in mental health. Front. Artif. Intell. 8:1465295. doi: 10.3389/frai.2025.1465295 PMC12315656 40702980

[ref28] Roca P. (2025). ¿Puede una mente artificial sanar una mente natural? Aplicaciones de la inteligencia artificial en psicología. In Ortiz J. M. Benguría J. (Coords.), Un nuevo conocimiento transversal: la inteligencia artificial aplicada (pp. 145–165). Valencia, Spain: Tirant lo Blanch

[ref29] Sahu M. Gupta R. Ambasta R. K. Kumar P. (2022). Artificial intelligence in mental health: opportunities, challenges, and future directions. Neurosci. Res. 179, 41–54. doi: 10.1016/j.neures.2022.05.008

[ref30] Shatte A. B. R. Hutchinson D. M. Teague S. J. (2019). Machine learning in mental health: a scoping review of methods and applications. Psychol. Med. 49, 1426–1448. doi: 10.1017/S0033291719000151, 30744717

[ref31] Spittal M. J. Guo X. A. Kang L. Kirtley O. J. Clapperton A. Hawton K. . (2025). Predicting suicide and self-harm in adolescents using machine learning: a population-based cohort study. PLoS Med. 22:e1004581. doi: 10.1371/journal.pmed.1004581 40934153 PMC12425223

[ref32] Spitzer R. L. Kroenke K. Williams J. B. W. Löwe B. (2006). A brief measure for assessing generalized anxiety disorder: the GAD-7. Arch. Intern. Med. 166, 1092–1097. doi: 10.1001/archinte.166.10.1092, 16717171

[ref33] Tavory T. (2024). Regulating AI in mental health: ethics of care perspective. J. Med. Ethics 50, 579–585. doi: 10.1136/jme-2024-109823, 39298759 PMC11450345

[ref34] Wang X. Zhou Y. Zhou G. (2025). The application and ethical implication of generative AI in mental health: systematic review. JMIR Mental Health 12:e70610. doi: 10.2196/70610, 40577783 PMC12254713

[ref35] Wolf R. M. Channa R. Liu T. Y. A. Zehra A. Bromberger L. Patel D. . (2024). Screening for diabetic retinopathy using a deep learning system. Nat. Commun. 15:421. doi: 10.1038/s41467-023-44676-z 38212308 PMC10784572

[ref36] World Health Organization. (2025). World mental health today: latest data. Available online at: https://www.who.int/publications/i/item/9789240113817 (Accessed December 29, 2025).

